# MRdb: a comprehensive database of univariate and multivariate Mendelian randomization with large-scale GWAS summary data

**DOI:** 10.1093/database/baaf054

**Published:** 2025-09-24

**Authors:** Qian Liu, Yujie Zhang, Houxing Li, Jiatong Li, Mengyu Xin, Rui Sun, Yifan Dai, Xinxin Shan, Yuting He, Borui Xu, Shangwei Ning, Peng Wang, Qiuyan Guo

**Affiliations:** Department of Gynecology, the First Affiliated Hospital of Harbin Medical University, 23 Youzheng Road, Harbin 150081, China; College of Bioinformatics Science and Technology, Harbin Medical University, 194 Xuefu Road, Harbin 150081, China; College of Bioinformatics Science and Technology, Harbin Medical University, 194 Xuefu Road, Harbin 150081, China; College of Bioinformatics Science and Technology, Harbin Medical University, 194 Xuefu Road, Harbin 150081, China; College of Bioinformatics Science and Technology, Harbin Medical University, 194 Xuefu Road, Harbin 150081, China; College of Bioinformatics Science and Technology, Harbin Medical University, 194 Xuefu Road, Harbin 150081, China; College of Bioinformatics Science and Technology, Harbin Medical University, 194 Xuefu Road, Harbin 150081, China; College of Bioinformatics Science and Technology, Harbin Medical University, 194 Xuefu Road, Harbin 150081, China; College of Bioinformatics Science and Technology, Harbin Medical University, 194 Xuefu Road, Harbin 150081, China; College of Bioinformatics Science and Technology, Harbin Medical University, 194 Xuefu Road, Harbin 150081, China; College of Bioinformatics Science and Technology, Harbin Medical University, 194 Xuefu Road, Harbin 150081, China; College of Bioinformatics Science and Technology, Harbin Medical University, 194 Xuefu Road, Harbin 150081, China; Department of Gynecology, the First Affiliated Hospital of Harbin Medical University, 23 Youzheng Road, Harbin 150081, China

## Abstract

Recent advancements highlight the importance of large-scale causal inference in elucidating disease mechanisms and guiding public health strategies. Mendelian randomization (MR) has become a cornerstone method for identifying causal relationships by leveraging genetic variants as instrumental variables. However, existing tools lack flexibility for multivariable analyses and fail to integrate diverse datasets effectively. To address these challenges, we introduce MRdb, a comprehensive database designed for conducting both univariable and multivariable MR analyses. MRdb encompasses 12 distinct categories of exposure data, including but not limited to 19 126 expression quantitative trait loci genes, 4907 plasma proteins, and 1400 plasma metabolites. Additionally, it integrates 48 507 disease outcomes sourced from FinnGen R10 and the IEU Open GWAS Project. MRdb offers robust data preprocessing features, including handling missing statistics, harmonizing datasets, and selecting instrumental variables to ensure high-quality analyses. Collectively, MRdb bridges the gaps in existing tools by integrating diverse datasets with user-friendly functionalities, empowering researchers to explore complex causal mechanisms.

## Introduction

Understanding complex disease mechanisms and informing public health interventions requires large-scale causal inference using extensive datasets [1]. These methods provide robust causal insights, minimize confounding factors, and clarify exposure–outcome interactions. Mendelian randomization (MR) uses genetic variations as instrumental variables to infer causal relationships between exposures and disease outcomes [2, 3]. MR exploits the random allocation of genetic variants to mitigate confounding factors and reverse causation in traditional observational studies [4]. In MR analysis, single nucleotide polymorphisms (SNPs), the most common type of genetic variation, serve as key instrumental variables [5]. Advances in genome-wide association studies (GWAS) have identified numerous SNPs linked to complex diseases, providing valuable insight into their genetic basis [6]. However, accurately linking these SNPs to disease outcomes remains a significant challenge [7].

The growth of biomedical big data is revealing an increasing number of causal links between genetic variants and diseases. Recent MR studies show that elevated plasma lipids, including triacylglycerol and phosphatidylinositol, increase the risk of multiple myeloma (MM), while certain phospholipids and sterol esters have a protective effect. These findings suggest that plasma lipid composition influences MM pathogenesis in complex ways [8]. Elevated levels of circulating metabolites such as methylmalonic acid are causally linked to an increased risk of rheumatoid arthritis (RA), emphasizing the role of metabolic dysregulation and the potential of novel biomarkers [9]. To further demonstrate the usefulness of MR in high-throughput analyses, multivariable MR (MVMR) studies have shown that various plasma lipid subtypes, such as low-density lipoprotein, high-density lipoprotein, and triglycerides, influence the risk of coronary artery disease (CAD) simultaneously. By considering the interdependencies among lipid traits, MVMR provides detailed information on their individual and combined roles in CAD pathogenesis. These studies demonstrate the utility of MR in causal inference and provide insights into the aetiology of complex diseases, aiding the development of therapeutic strategies. Multiple databases and data catalogues have been developed to support such research, including DMRdb, PhenoScanner, MR-Base, and the GWAS Catalog [10–13]. These resources are essential for studying the relationship between genetic variations and phenotypes. With the continuous generation of biomedical big data, the ever-increasing volume of data enables us to conduct more comprehensive and in-depth analyses. Thus, we created MRdb, a comprehensive database tailored for univariable and multivariable MR studies. MRdb integrates high-quality GWAS data, offering researchers a robust tool for comprehensive and flexible causal inference analyses. As a fully integrated platform, MRdb is regularly updated with the latest genetic data and methods, serving as a key resource for investigating causal links between therapeutic targets and disease phenotypes.

## Materials and methods

### Data acquisition and curation

To develop the MRdb database, we collected exposure data from 12 high-quality GWAS data sources, encompassing a range of biological indicators, such as plasma proteins, metabolites, and immune cell types. Due to the variability in data formats across these sources, we first normalized variables across all datasets into a consistent format. All genomic locations were uniformly converted to the GRCh38 genome reference version, ensuring that data from disparate studies could be compared and analysed within a unified genomic framework. This standardization included formatting fields for key parameters such as SNP ID (rsID), effect allele (effect_allele), other allele (other_allele), effect allele frequency (effect_allele_frequency), effect size (beta), standard error (standard_error), and *P*-value (p_value). By establishing this uniform structure, we ensured comparability across datasets, providing a robust foundation for subsequent analyses. To maintain data integrity and mitigate the impact of missing values, we employed statistical imputation methods based on available summary statistics [10]. The specific imputation approaches are detailed below:

1. Estimation of beta values: For data that included odds ratios (OR) but lacked corresponding beta values, we derived beta estimates using the natural logarithm transformation:
  \begin{eqnarray*}
\beta = {\mathrm{ln}}\, ({\mathrm{ OR}}).
\end{eqnarray*}
  This formula allows for a consistent and reliable effect size estimation in the absence of direct beta values, enhancing the coherence and robustness of the analysis results.
2. Estimation of SE values: When data contained beta values and *P*-values but lacked standard errors (SE), we calculated SE using the following formula:
  \begin{eqnarray*}
{\mathrm{ SE}} = \sqrt {\frac{{{{\beta }^2}}}{{x_{1,{\mathrm{ upper}}}^2\left( P \right)}}}.
\end{eqnarray*}
  Here, $\mathrm{ SE}{\mathrm{\ }}$ represents the SE, $\beta {\mathrm{\ }}$denotes the effect size estimate, $P{\mathrm{\ }}$refers to the *P*-value, and $x_{1,{\mathrm{ upper}}}^2( P ){\mathrm{\ }}$represents the upper quantile chi-square value with one degree of freedom corresponding to the given *P*-value. This approach enables indirect $\mathrm{ SE}{\mathrm{\ }}$ estimation when direct $\mathrm{ SE}$ data are unavailable, thereby providing a solid basis for subsequent statistical analyses.
3. Estimation of *P*-value: Given available β and SE values, we derived *P*-values using the *Z*-test approach. This procedure ensures a statistically robust way to determine the significance of effect sizes.
  \begin{eqnarray*}
{P} = 2\ \times \ {\mathrm{\Phi }}\left( { - \left| {\frac{\beta }{{\mathrm{ SE}}}} \right|} \right),
\end{eqnarray*}
  where ${\mathrm{\Phi \ }}$ refers to the cumulative distribution function of the standard normal distribution. By calculating the *Z*-score as the absolute value of the ratio $\frac{\beta }{{\mathrm{ SE}}}$ and subsequently deriving the *P*-value through a two-tailed test, this approach provides a reliable significance assessment for each effect size.

Following exposure data standardization, we implemented quality control procedures. First, we filtered SNPs using a *P*-value threshold (*P* < 1e-5) to retain only those significantly associated with exposures. Next, we performed LD clumping via the ld_clump function in the ieugwasr R package (v1.0.3) [12], leveraging a European reference panel with parameters set to *r*^2^ = 0.001 and a 10 000 kb window to ensure SNP independence. To mitigate weak instrument bias, we retained only SNPs with *F*-statistics > 10. These preprocessing steps enhance the validity and robustness of causal inference in subsequent MR analyses.

### Univariable MR analysis

In MRdb, univariable MR analysis serves as a fundamental approach for inferring causal relationships between a single exposure and a single disease outcome. This method estimates the causal effect between exposure and outcome by leveraging multiple instrumental variables. The process entails rigorous selection of instrumental variables, precise effect estimation, comprehensive sensitivity analysis, and conclusive causal inference, ensuring a robust framework for assessing causality in MR studies.

MRdb offers multiple methods for univariable MR analysis, primarily based on the TwoSampleMR R package (v0.6.16). These methods include the Inverse Variance Weighted (IVW) [14], MR-Egger regression [15], and the Wald ratio method [16], among others. Each of these techniques provides a unique approach to estimate causal effects, with IVW being widely used for its efficiency in synthesizing multiple SNP estimates, while MR-Egger regression and the Wald ratio method offer alternatives that account for potential biases and instrument strength.

To ensure robust univariable MR results, MRdb offers comprehensive sensitivity analysis tools and heterogeneity testing. Users can apply methods such as MR-Egger regression and MR-PRESSO to evaluate horizontal pleiotropy [17]. Heterogeneity is assessed via Cochran’s *Q* test, which quantifies inter-SNP variability in effect sizes [18]. Significant heterogeneity may indicate violations of MR assumptions, prompting further investigation. Additionally, leave-one-out analysis is available to identify influential SNPs driving heterogeneity, flagging potential pleiotropy or bias [19].

### Multivariable MR analysis

In MVMR analysis, the MRdb platform leverages the MVMR R package (v0.4.1) to conduct integrative analyses of multiple exposure factors with a single outcome variable, addressing the limitations of traditional univariable approaches in handling complex causal relationships [20]. This functionality is designed to help researchers manage potential interactions and confounding effects among exposure factors, thereby enabling a more nuanced exploration of causality.

The MVMR analysis framework in MRdb supports two primary analytical approaches: (i) IVW method, which estimates the combined effect of multiple exposure factors on an outcome variable through a weighted linear regression model. (ii) MR-Egger method, which, unlike IVW, permits a non-zero intercept and thus can accommodate potential horizontal pleiotropy among exposure factors. Based on these two methods, users can adjust the Orientate parameter to specify the exposure variable direction of genetic association, typically defaulting to the first exposure variable for forward effect analysis. MRdb’s multivariable analysis module also includes LASSO feature selection, enabling users to identify the most representative instrumental variables to streamline and enhance model precision. The MR-PRESSO correction option aids in adjusting for horizontal pleiotropy, minimizing potential pleiotropic bias. Through these flexible parameter settings, MRdb ensures robustness and reliability in multivariable MR analysis outcomes.

To assess heterogeneity in MVMR analysis, MRdb provides the Cochran *Q* test, which detects inconsistencies among instrumental effects. A high *Q* statistic with a significant *P*-value may indicate biased causal estimates. The *F* statistic is also employed to evaluate the strength of the instruments, helping to identify weak SNPs. To further validate robustness, MRdb offers sensitivity tools such as leave-one-out analysis, funnel plots, forest plots, and density plots. MRdb also provides a range of comprehensive causal estimates, including effect sizes, SE, and *P*-values for each exposure. Results from the IVW and MR-Egger methods are accompanied by tests for heterogeneity and the *F* statistic to ensure robustness. MRdb also supports flexible parameter settings and sensitivity analyses, enabling users to investigate the combined effects of exposures and complex causal networks.

### Data records and code availability

The datasets can be obtained from the download page of MRdb (http://www.bio-server.cn/MRdb/Download.html). The exposure data encompasses 12 distinct categories. These include 19 126 eQTL genes, 4907 plasma proteins, 1400 plasma metabolites, 731 immune cell types, 486 blood metabolites, 597 skin microbial components, 412 gut microbial components, 179 plasma lipids, 91 circulating proteins, 74 blood cell types, 41 inflammatory factors, and 8 thyroid hormones. The outcome data comprises 2408 phenotypes retrieved from FinnGen R10 and processed against each of the 12 exposure categories, yielding 28 896 outcome files in total. All data underwent standardized pre-processing procedures, such as quality control, data harmonization, and file formatting, to ensure consistency and reliability for subsequent MR analyses. The R codes used to generate the datasets in MRdb were shared on GitHub (https://github.com/LiuQian12138/MRdb).

### Database construction

MRdb’s online platform is developed using Java Server Pages, with data processing scripts written in Java. The web service is hosted and maintained on an Apache Tomcat web server. MRdb is compatible with major modern browsers, including Microsoft Edge, Google Chrome, Firefox, and Safari. Datasets in MRdb are stored and managed via the open-source MySQL database system. The web interface can be accessed at http://www.bio-server.cn/MRdb/.

## Results

### Database content and function

**Figure 1.**
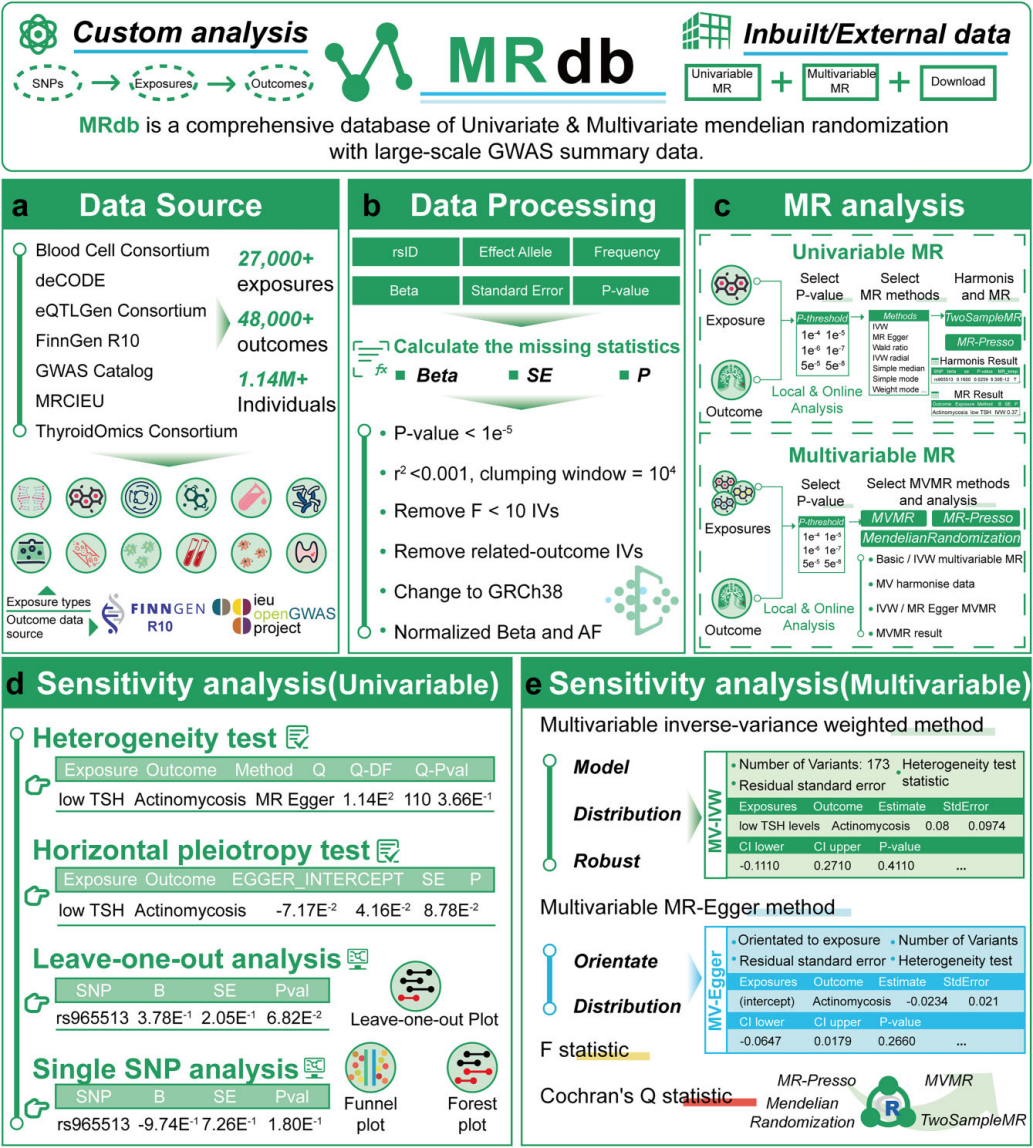
The systemic overview of MRdb. (a) MRdb integrates 12 types of exposure data, including eQTL genes, plasma proteins, and metabolites, alongside 48 507 disease outcomes from GWAS datasets such as FinnGen R10 and the IEU Open GWAS Project. (b) Data preprocessing includes handling missing statistics, harmonizing datasets, normalizing allele frequencies, and selecting valid instrumental variables to ensure high-quality analyses. (c) MRdb provides tools for univariable and multivariable MR analyses, offering outputs such as causal estimates, *P*-values, and visualizations to support causal inference. (d) Sensitivity analysis for univariable MR includes heterogeneity testing, pleiotropy analysis, leave-one-out validation, and single SNP analysis with forest and funnel plots. (e) For multivariable MR, MRdb supports advanced metrics such as *F*-statistics and Cochran’s *Q* test to evaluate model robustness and reliability.

We systematically collected and integrated high-quality GWAS datasets across diverse areas. We integrated exposure data covering 19 126 expression quantitative trait loci (eQTL) genes [21], 4907 plasma proteins [22], 1400 plasma metabolites [23], 731 immune cell types [24], 486 blood metabolites [25], 597 skin microbiome components [26], 412 gut microbiome components [27], 179 plasma lipids [28], 91 circulating proteins [29], 74 blood cell types [30], 41 inflammatory cytokines [31], and 8 thyroid hormones [32], sourced from reputable consortia such as eQTLGen, Blood Cell Consortium, deCODE, MRCIEU, and Thyroid Omics. The outcome datasets were mainly obtained from FinnGen R10 (2408 traits, ~5%) and the IEU OpenGWAS Project (46 099 traits, ~95%), reflecting a comprehensive coverage of disease endpoints and complex phenotypes [12, 33]. In total, MRdb includes over 27 000 exposures and 48 000 outcomes from more than 1.14 million individuals (Fig. 1a, Supplementary Tables S1 and S2). To ensure analytical rigour and data consistency, all GWAS summary statistics were subjected to standardized preprocessing, including computation of missing effect sizes and standard errors, harmonization to GRCh38, normalization of beta values and allele frequencies, removal of SNPs with weak instrument strength, and exclusion of outcome-related or correlated instruments (Fig. 1b). MRdb supports both univariable and multivariable MR analyses using TwoSampleMR, MVMR, and MR-PRESSO frameworks (Fig. 1c). For univariable MR, users can conduct IVW, MR-Egger, weighted median, and MR-PRESSO analyses. MRdb provides comprehensive sensitivity analyses, including heterogeneity testing, horizontal pleiotropy testing through the MR-Egger intercept, leave-one-out analysis to assess the influence of individual SNPs, and single SNP effect analysis using forest and funnel plots (Fig. 1d). For multivariable MR, sensitivity diagnostics include multivariable IVW and MR-Egger regression with model evaluation metrics such as residual standard error, *F*-statistics, orientation analysis, and multivariate *Q*-statistics to rigorously assess model robustness and instrument validity (Fig. 1e).

### Usage note

Through MRdb, users can access these datasets and flexibly select analytical methods based on research needs. To further enhance data reliability, all SNPs undergo linkage disequilibrium (LD) screening and standardization, ensuring data independence and consistency. Users can employ MRdb for both univariable MR and MVMR analyses to explore potential causal impacts of single or multiple exposures on outcomes. The platform also offers sensitivity analysis tools, such as MR-Egger regression and IVW methods, for detecting and adjusting pleiotropic bias. Tools like MR-PRESSO are available to correct pleiotropy-induced biases, improving the precision and robustness of causal inference. Visualization tools, including funnel plots and forest plots, are provided to facilitate intuitive understanding of heterogeneity and potential pleiotropic bias in the data. Users can download datasets from the MRdb homepage (http://www.bio-server.cn/MRdb) and customize analyses as needed. MRdb offers researchers a flexible and reliable causal inference tool, ideal for multi-level analysis of complex genomic data and providing robust support for MR studies.

**Figure 2.**
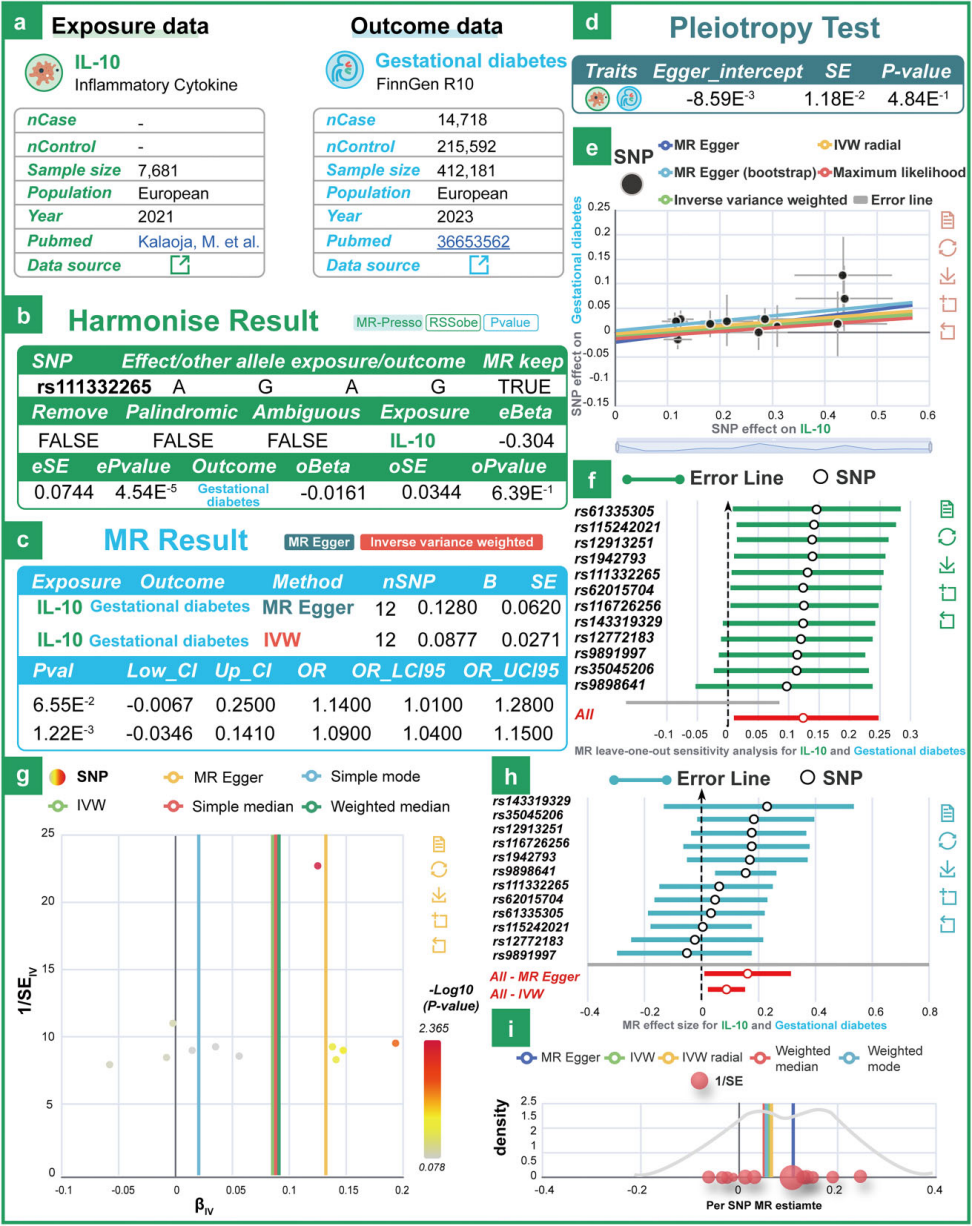
Examples of technical validation of the data. (a) The details of the exposure (IL-10) and outcome (gestational diabetes) datasets used in MR analysis. (b) The harmonization results for exposure and outcome data. (c) The MR results using two statistical methods: MR-Egger and IVW. (d) The pleiotropy assessment examines the presence of directional bias in the genetic instruments. (e)–(i) Leave-one-out sensitivity plot, funnel plot, forest plot, and density plot between IL-10 and gestational diabetes.

To further validate causal associations between exposure and outcome variables, MRdb applies both univariable and multivariable MR frameworks (2SMR and MVMR) with stringent instrumental variable selection, ensuring that selected SNPs have an *F*-statistic greater than 10. To further validate our dataset, we investigated the relationship between interleukin-10 (IL-10) and gestational diabetes mellitus (GDM). Previous studies have investigated the association between the expression of IL-10 and GDM using immunohistochemistry method. However, the current scientific evidence does not directly establish a statistically significant association between IL-10 gene expression and the pathogenesis of GDM. Based on MRdb, we estimated a causal effect between IL-10 and GDM (Fig. 2a and b; OR: 1.09; 95% CI: 1.01–1.28; *P* = 1.22e-3). Using IVW and MR-Egger regression methods, MRdb can identify and adjust for potential horizontal pleiotropy, ensuring the reliability of the causal inference results (Fig. 2c and d). To address data heterogeneity issues, MRdb incorporates a range of testing and integration methods for heterogeneity. Tools such as the Cochran *Q* test, funnel plot, and forest plot are used to effectively detect and visualize heterogeneity levels across different datasets. MRdb also employs various sensitivity analysis tools, including leave-one-out analysis, to identify and mitigate the impact of outliers, thereby enhancing the reliability of the results (Fig. 2e–i). These tools, combined with data integration strategies, enable researchers to comprehensively analyse causal relationships between exposure variables and outcomes while identifying potential biases and data noise.

## Discussion and conclusion

Large-scale causal inference with extensive datasets is critical for dissecting complex disease mechanisms and guiding public health interventions. Leveraging genetic variants as instrumental variables, MR enables robust causal inference by minimizing confounding and clarifying exposure–outcome relationships. The exponential growth of biomedical big data, particularly in genomics and clinical phenomics, has uncovered a burgeoning landscape of causal links between genetic variants, molecular phenotypes, and complex diseases. To facilitate robust causal inference in this era of data-driven research, multiple specialized databases have emerged [10–13]. While these databases are all designed to improve MR analysis, they differ in their focus, and each has its own strengths. For example, PhenoScanner allows users to explore SNP associations with diverse phenotypes and gene expression levels. MR-Base specializes in MR analysis and supports univariable and limited multivariable approaches. The GWAS Catalog compiles extensive GWAS results. DMRdb is a disease-centric MR database designed to systematically assess the causal relationships between diseases, genes, proteins, CpG sites, metabolites, and other diseases. MRdb is a comprehensive univariate and multivariate MR database featuring large-scale GWAS summary data, including eQTL genes, plasma proteins/metabolites, immune cells, microbiome components, circulating proteins, blood cells, inflammatory cytokines, and outcomes from FinnGen R10 and the IEU Open GWAS Project. We believe that the complementary strengths of MRdb and other databases in terms of data coverage and methodological approaches will collectively advance the field of MR by providing more robust causal inference services.

## Supplementary Material

baaf054_Supplemental_File
