# Supplementary material for: MRdb: a comprehensive database of univariate and multivariate Mendelian randomization with large-scale GWAS summary data
Source: Database (Oxford). 2025 Sep 24;2025:baaf054. doi: 10.1093/database/baaf054 (PMC12462376; doi:10.1093/database/baaf054)
Supplement: baaf054_Supplemental_File [file baaf054_supplemental_file.pdf]

Supplementary Information for

MRdb: a comprehensive database of Univariate & Multivariate mendelian randomization with large-scale GWAS summary data

This file includes:

Supplementary Tables (Table S1-S2)

Table S1. The data content of MRdb and its comparison with other databases.

	**MRdb**	**DMRdb**	**MR-Base**
Exposure: IL-10			
Sample Size	7,681	3,301	NA
Number of SNPs	9,500,544	10,534,735	NA
Outcome: Gestational diabetes			
Sample Size	412,181	412,181	NA
Ncase/Ncontrol	14718/215592	14718/215592	NA
Exposure type	12	5	6
Thyroid Omics	8	0	0
Microbiome	702	0	0
Immune Omics	846	0	0
mQTL	2,065	825	725
pQTL	4,998	2,564	47
eQTL	19,126	16,238	27,094
Outcome datasets	48,507	38,267	46,099
Data source	FinnGen R10, GWAS Catalog, IEU database, Other specialized data websites	FinnGen R9,FinnGen R10, GWAS Catalog, IEU database, UK Biobank	IEU database
Tools			
Univariable MR	√	√	√
Multivariable MR	√	×	×
Custom Analysis	√	×	√
Disease GWAS	×	√	×

Table S2. Statistics of outcome datasets in MRdb.

Exposure categories	Datasets	individuals	Consortium
Thyroid Omics	8	271,040	ThyroidOmics Consortium
Inflammatory cytokines	41	8,239	Cytokines GWAS
Blood cells	74	746,667	Blood Cell Consortium
Circulating proteins	91	14,824	GWAS Catalog
Plasma lipidome	179	7,174	GWAS Catalog
Skin microbiota	290	597	GWAS Catalog
Dutch gut microbiota	412	7,738	GWAS Catalog
Blood metabolites	486	7,824	GWAS Catalog
Immune cells	731	3,757	GWAS Catalog
Plasma metabolome	1,400	8,299	GWAS Catalog
Plasma proteome	4,907	35,559	deCODE
eQTLGen (cis-eQTLs)	19,126	31,684	eQTLGen
